# Efficacy and safety of post-discharge oral nutritional supplements for patients with gastric cancer undergoing gastrectomy: a meta-analysis of randomized controlled trials

**DOI:** 10.3389/fnut.2024.1488054

**Published:** 2025-01-14

**Authors:** Qiuman Liang, Siyi Wang, Beibei Wang, Yanyan Hong

**Affiliations:** ^1^Nanjing Hospital of Chinese Medicine Affiliated to Nanjing University of Chinese Medicine, Nanjing, Jiangsu, China; ^2^School of Nursing, Nanjing University of Chinese Medicine, Nanjing, Jiangsu, China; ^3^School of Elderly Care Services and Management, Nanjing University of Chinese Medicine, Nanjing, Jiangsu, China

**Keywords:** gastric cancer, gastrectomy, meta-analysis, ONS, systematic review

## Abstract

**Objectives:**

To report the first and largest systematic review and meta-analysis of randomized controlled trials (RCT) to evaluated the efficacy and safety of post-discharge oral nutritional supplements (ONS) for patients with gastric cancer undergoing gastrectomy.

**Design:**

Systematic review and meta-analysis.

**Eligibility criteria for selecting studies:**

RCT which evaluated the efficacy and/or safety of post-discharge ONS for patients with gastric cancer undergoing gastrectomy.

**Data sources:**

We conducted a systematic literature retrieval via PubMed, Embase, Web of Science, and Cochrane until April, 2023 for relevant RCTs.

**Data analysis:**

Outcomes of meta-analysis included absolute change of body weight, % change of body weight, absolute change of body composition, absolute change of laboratory parameters and adverse events. All the relevant data were analyzed by Review Manager 5.4.1 and Stata 15.1.

**Results:**

5 RCTs including 1,586 patients (804 in ONS group versus 782 in control group) were included for meta-analysis. The two groups were comparable in age, gender (male), weight at baseline, BMI at baseline, albumin at baseline, and hemoglobin at baseline. Meta-analysis revealed a significant lower absolute body weight loss (WMD: 0.75; 95% CI: 0.11, 1.40; *p* = 0.02) and % body weight loss (WMD: 1.15; 95% CI: 0.20, 2.11; *p* = 0.02) in the ONS group compared with the control (regular diet/dietary advice) group. Moreover, this study did not observe a significant difference between the two groups for adverse events rate (RR: 1.11; 95% CI: 0.81, 1.53; *p* = 0.52).

**Conclusion:**

ONS was significantly effective and safe in improving postoperative weight loss for patients with gastric cancer undergoing gastrectomy.

**Systematic review registration:**

Identifier, CRD42023414678, https://www.crd.york.ac.uk/PROSPERO/.

## Introduction

Gastric cancer is a particularly common malignant tumor of digestive tract, which has become one of the main causes of cancer-related death worldwide and has caused great damage to human health (1, 2). At present, although chemotherapy, immunotherapy have made great development (3, 4), gastrectomy is still the main and most effective treatment for gastric cancer (5). However, due to various risk factors such as reduced stomach volume and food intake after gastrectomy, postoperative chemotherapy, postoperative gastrointestinal symptoms, the incidence of malnutrition in patients with gastric cancer after surgery is very high and their nutritional status would deteriorate gradually (6). A large number of studies have suggested that malnutrition is significantly associated with a variety of adverse outcomes in postoperative gastric cancer patients, including higher morbidity and mortality, lower chemotherapy tolerance and poorer survival condition (7–9). Therefore, active nutritional support is of great significance for improving the nutritional status and tumor prognosis of postoperative patients with gastric cancer (10).

Oral nutritional supplements (ONS) are currently the best way of nutritional support treatment, which can provide supplementary energy and nutrition for special groups such as gastric cancer patients after surgery (11). Despite a large number of clinical studies and systematic reviews have reported the application of ONS in patients undergoing gastrectomy, their conclusions are inconsistent (10, 12–19). One of the causes for these differences is that there is no established ONS scheme. In addition, patient compliance with ONS may be another critical factor, which is strongly influenced by nutritional consultations with surgeons, dietitians, and pharmacologists. Furthermore, although some clinical studies have observed the effectiveness of ONS on short-term nutrition-related outcomes, such as reduced postoperative infection rate and shorter hospital stay, the nutritional efficacy of ONS on medium- and long-term outcomes (such as weight loss of gastric cancer patients after gastrectomy) have not been fully demonstrated (17).

Although the meta-analysis of Chen et al. (19) and Choi et al. (20) showed that preoperative or perioperative use of ONS could significantly reduce postoperative inflammatory response and improve immune system function and nutritional status in patients with gastric cancer resection. Existing evidence-based studies have failed to clarify whether long-term regular use of ONS after surgery can improve the nutritional status and prognosis of discharged gastric cancer patients after surgery. In addition, previous meta-analyses did not discuss the impact of follow-up after the use of ONS and surgical methods for gastric cancer on the long-term efficacy of ONS, and there was a lack of standard GRADE evidence recommendation. Therefore, we report the first and largest systematic review and meta-analysis of randomized controlled trials (RCTs) to evaluate the efficacy and safety of long-term regular ONS in patients undergoing gastrectomy after discharge, and to make evidence recommendations through GRADE ratings to provide evidence-based evidence for clinical use of ONS to improve long-term postoperative nutritional status in patients with gastric cancer.

## Methods

### Patient and public involvement

No patient involved.

### Registration

This meta-analysis was performed according to the PRISMA (Preferred Reporting Items for Systematic Reviews and Meta-Analysis) 2020 statement (21) and has been prospectively registered in the PROSPERO (CRD42023414678).

### Literature search

We conducted a systematic literature search via PubMed, Embase, Web of Science, and Cochrane up to April, 2023 for RCT that evaluated the efficacy and safety of post-discharge ONS for patients with gastric cancer undergoing gastrectomy. We searched the literature through the following terms: “stomach neoplasms,” “gastrectomy,” “oral nutritional supplements,” and “ONS.” The detailed search strategies are as follows: (((Oral nutritional supplements) OR (ONS)) AND ((Gastrectomies) OR (“Gastrectomy”[Mesh]))) AND (((((((((((((((((((Neoplasm, Stomach[Title/Abstract]) OR (Stomach Neoplasm[Title/Abstract])) OR (Neoplasms, Stomach[Title/Abstract])) OR (Gastric Neoplasms[Title/Abstract])) OR (Gastric Neoplasm[Title/Abstract])) OR (Neoplasm, Gastric[Title/Abstract])) OR (Neoplasms, Gastric[Title/Abstract])) OR (Cancer of Stomach[Title/Abstract])) OR (Stomach Cancers[Title/Abstract])) OR (Gastric Cancer[Title/Abstract])) OR (Cancer, Gastric[Title/Abstract])) OR (Cancers, Gastric[Title/Abstract])) OR (Gastric Cancers[Title/Abstract])) OR (Stomach Cancer[Title/Abstract])) OR (Cancer, Stomach[Title/Abstract])) OR (Cancers, Stomach[Title/Abstract])) OR (Cancer of the Stomach[Title/Abstract])) OR (Gastric Cancer, Familial Diffuse[Title/Abstract])) OR (“Stomach Neoplasms”[Mesh])). Furthermore, we manually screened the bibliography lists of all included RCTs. Two authors retrieved and assessed eligible articles independently. Any differences in literature retrieval were resolved by discussion.

### Inclusion criteria

Articles were eligible when meeting the following PICOS standards: P: patients with gastric cancer undergoing gastrectomy; I: the long-term (continuous supplementation for ≥1 month) post-discharge supplementation of ONS; C: usual postoperative diet, or dietary advice; O: absolute change of body weight, % change of body weight (the value of weight loss as a percentage of baseline weight), change of skeletal muscle mass, change of body fat mass, change of albumin, change of total protein, change of total cholesterol, change of hemoglobin and adverse events; S: RCT. In addition, studies were eligible if there was complete data to analyze risk ratio (RR), weighted mean difference (WMD) or standard mean difference (SMD).

### Exclusion criteria

We excluded study protocols, unpublished studies, non-original studies (including letters, comments, abstracts, correction, and reply), non-RCT studies, studies without sufficient data, and reviews. In addition, we excluded studies in which short-term ONS administration was initiated before or perioperatively.

### Data abstraction

Data abstraction was conducted by two authors severally. Any differences were settled by another author. We abstracted following information from eligible RCTs: first author name, published year, research period, study region, study design, registration number, type of gastrectomy, intervention, control, sample size, age, gender, follow-up time, weight at baseline, body mass index (BMI) at baseline, albumin at baseline, hemoglobin at baseline, absolute change of body weight, % change of body weight, change of skeletal muscle mass, change of body fat mass, change of albumin, change of total protein, change of total cholesterol, change of hemoglobin, and adverse events. If the continuous data in the article was presented as median plus range or median plus interquartile range (IQR), we reanalyzed the mean ± standard deviation (SD) via the methods reported by Wan et al. and Luo et al. (22, 23). If the research data is insufficient, corresponding authors were contacted for full data if available.

### Quality evaluation

The quality assessment of eligible RCTs was conducted using the Risk of Bias 2 (RoB 2) tool. The following items were assessed as a possible source of bias: process of randomization, deviations from intended interventions, missing outcome data, measurement of outcome, and reported results selection. For each item, multiple standardized questions are answered with ‘yes’, ‘probably yes’, ‘probably no’, ‘no’, and ‘no information’. Then, based on these answers, the risk of bias for each item was judged as ‘low risk’, ‘some concerns’, or ‘high risk’ (24). Two authors severally assessed the quality of all included studies, and any disagreement was settled by discussion.

### Statistical analysis

Meta-analysis was conducted in Review Manager 5.4.1 edition. For continuous data, the WMD or SMD was used for data synthesis, and the RR were used for the synthesis of dichotomous data. Each metric was presented with 95% confidential intervals (CIs). The chi-squared (χ^2^) test (Cochran’s *Q*) and inconsistency index (*I*^2^) were applied for the evaluation of the heterogeneity of each outcome (25). χ^2^ *p* value less than 0.1 or *I*^2^ more than 50% were regarded as high heterogeneity. The random-effects model was applied to calculate the total WMD, SMD or RR. In addition, we performed subgroup analyses for efficacy outcomes with two or more included studies to evaluate the possible confounders, if data were sufficient. Besides, we conducted sensitivity analysis to assess the influence of every included RCT on the total WMD, SMD or RR for results with more than 2 included studies and significant heterogeneity. Moreover, we assessed the potential publication bias by producing funnel plots through Review Manager 5.4.1 edition as well as through performing Egger’s regression tests (26) through Stata 15.1 edition (Stata Corp, College Station, Texas, United States). *p* value <0.05 was considered as statistically significant publication bias.

### Quality evaluation of evidence

According to the Grading of Recommendations, Assessment, Development and Evaluation (GRADE), evidence of absolute change of body weight, % change of body weight and adverse events were evaluated through the follow items: risk of bias, inconsistency, indirectness, imprecision, publication bias, plausible confounding, magnitude of effect, and dose–response gradient. Finally, each outcome was graded as “high,” “moderate,” “low” or “very low” quality to draw conclusions (27).

## Results

### Literature retrieval, study characteristics, and baseline

**Figure 1:**
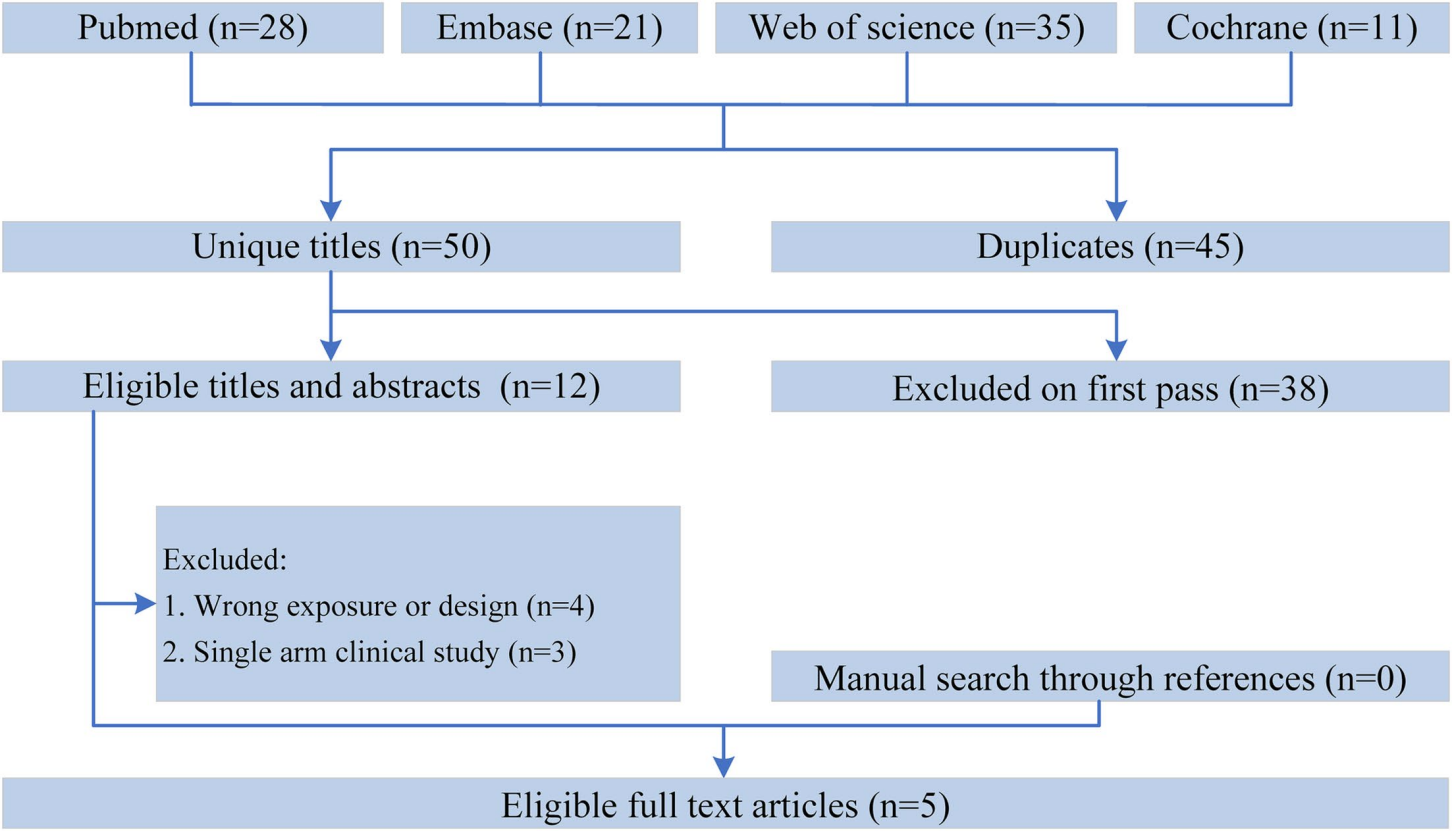
Flowchart of the systematic search and selection process.

Figure 1 shows the flowchart of the literature retrieval and selection process. A total of 95 related studies in PubMed (*n* = 28), Embase (*n* = 21), Web of Science (*n* = 35), and Cochrane (*n* = 11) were identified via systematically literature search. After removing duplicate studies, a total of 50 titles and abstracts were evaluated. Eventually, 5 RCTs including 1,586 patients (804 in ONS group versus 782 in control group) were included for meta-analysis (10, 15–18). Table 1 presents the characteristics of each eligible RCT. The two groups were comparable in age (WMD: 0.05; 95% CI: −1.00, 1.09; *p* = 0.93), gender (male; RR: 1.00; 95% CI: 0.93, 1.07; *p* = 1.00), weight at baseline (WMD: −0.43; 95% CI: −1.44, 0.58; *p* = 0.40), BMI at baseline (WMD: 0.01; 95% CI: −0.30, 0.32; *p* = 0.96), albumin at baseline (SMD: 0.00; 95% CI: −0.10, 0.10; *p* = 0.98), and hemoglobin at baseline (SMD: −0.01; 95% CI: −0.11, 0.09; *p* = 0.82; Table 2).

**Table 1:** Baseline characteristics of include RCTs.

Authors	Study period	Country	Study design	Registration number	Type of gastrectomy	Intervention	Control	Patients (n)	Follow-up
								ONS/Control	
Hatao 2017	2010–2012	Japan/China	RCT	UMIN000004650	Distal gastrectomy (*n* = 73) and total gastrectomy (*n* = 40)	Concentrated Liquid Diet ANOM® (Otsuka, Japan)	Usual postoperative diet (1400–1,600 kcal/day)	64/49	12 weeks
Imamura 2016	2011–2012	Japan	RCT	UMIN000008056	Distal gastrectomy (*n* = 73) and total gastrectomy (*n* = 38)	Elental®, Ajinomoto Pharmaceuticals, Tokyo, Japan	Regular diet	58/53	8 weeks
Meng 2021	2017–2018	China	RCT	ChiCTR2000029708	Distal gastrectomy (*n* = 233) and total gastrectomy (*n* = 104)	Nutren® Optimum (Nestle Health Science, Switzerland) at a 500 mL daily dosage	Dietary advice	171/166	3 months
Miyazaki 2021	2013–2017	Japan	RCT	UMIN000011919	Distal gastrectomy (*n* = 641), total gastrectomy (*n* = 300), and proximal gastrectomy (*n* = 62)	400 mL/day (400 kcal/day) Racol® NF	Regular diet	500/503	12 months
Toyomasu 2019	2011–2014	Japan	RCT	NA	Distal gastrectomy (*n* = 14) and total gastrectomy (*n* = 8)	One pack of Elental® per day	Regular diet	11/11	2 months

**Table 2:** Demographics and clinical characteristics of included studies.

Outcomes	Studies	No. of patients	WMD/SMD or RR	95% CI	*p*-value	Heterogeneity			
		ONS/Control				Chi^2^	df	*p*-value	*I*^2^ (%)
Age (years)	5	804/782	0.05	[−1.00, 1.09]	0.93	4.05	4	0.40	1
Gender (male)	5	804/782	1.00	[0.93, 1.07]	1.00	1.68	4	0.79	0
BMI (kg/m^2^)	4	793/771	0.01	[−0.30, 0.32]	0.96	2.76	3	0.43	0
Body weight (kg)	4	793/771	-0.43	[−1.44, 0.58]	0.40	1.78	3	0.62	0
Albumin (g/L or g/dL)	4	740/733	0.00	[−0.10, 0.10]	0.98	1.33	3	0.72	0
Hemoglobin (g/L or g/dL)	4	740/733	-0.01	[−0.11, 0.09]	0.82	3.46	3	0.33	13

BMI, body mass index; ONS, oral nutritional supplements; WMD, weighted mean difference; SMD, standard mean difference; RR, risk ratio; CI, confidence interval.

### Risk of bias assessment

Overall, one RCTs (15) was rated as low risk and four RCTs (10, 16–18) were rated as of some concerns. Among them, all RCTs adopted the correct random allocation method. However, due to the non-blind study design, deviations from intended intervention in four RCTs (10, 16–18) were rated as of some concerns. In addition, the measurement of the outcome in three RCTs (10, 16, 17) was rated as of some concerns because the methodology of the outcome evaluation was not reported in detail. The remaining entries are all low risk. Details of the quality evaluation for all included RCTs are shown in Supplementary Figure S1.

### Absolute change of body weight

**Figure 2:**
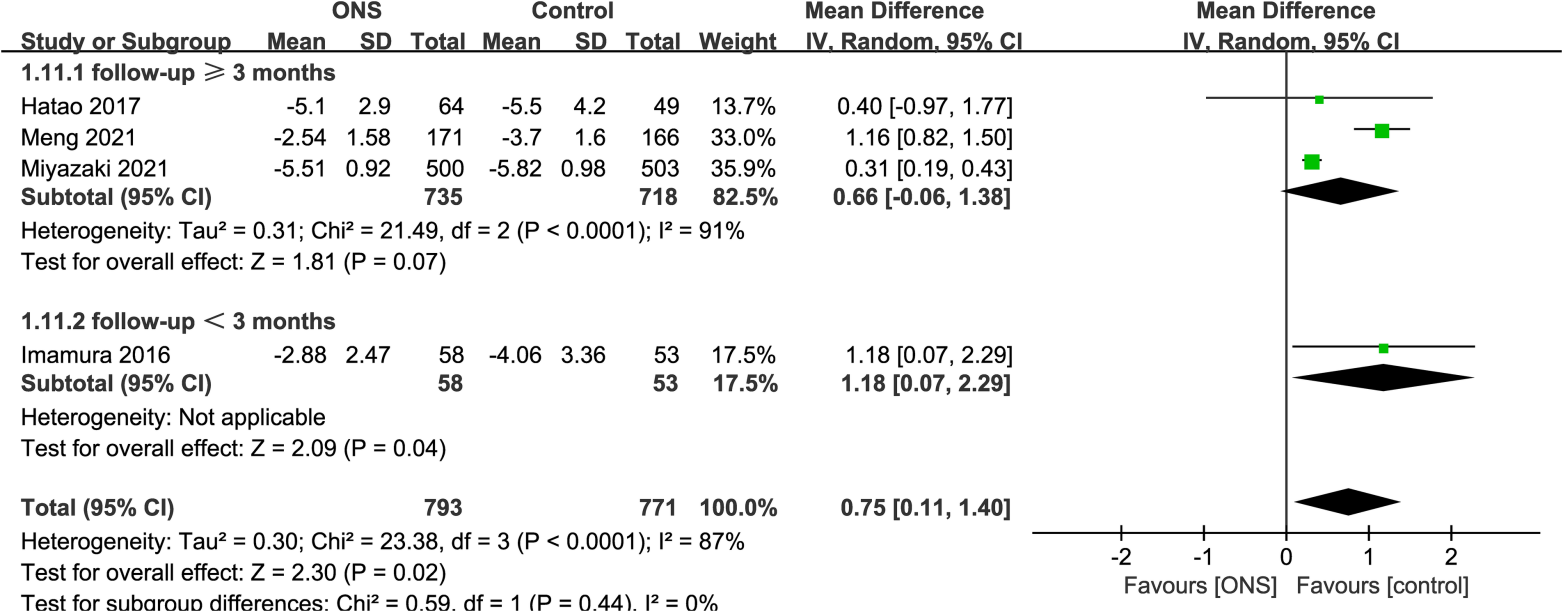
Forest plots of absolute change of body weight.

Results of absolute change of body weight were synthesized from 4 RCTs including 1,564 patients (793 ONS versus 771 control) (10, 15–17). Meta-analysis revealed a significant lower absolute body weight loss in the ONS group (WMD: 0.75; 95% CI: 0.11, 1.40; *p* = 0.02) with a significant heterogeneity (*I*^2^ = 87%, *p* < 0.0001; Figure 2). Subgroup analysis found that there was still a significant difference in study with a follow-up time of less than 3 months (WMD: 1.18; 95% CI: 0.07, 2.29; *p* = 0.04) (10), while the significance disappeared in studies with a follow-up time of ≥3 months (WMD: 0.66; 95% CI: −0.06, 1.38; *p* = 0.07; Figure 2) (15, 16).

### % change of body weight

**Figure 3:**
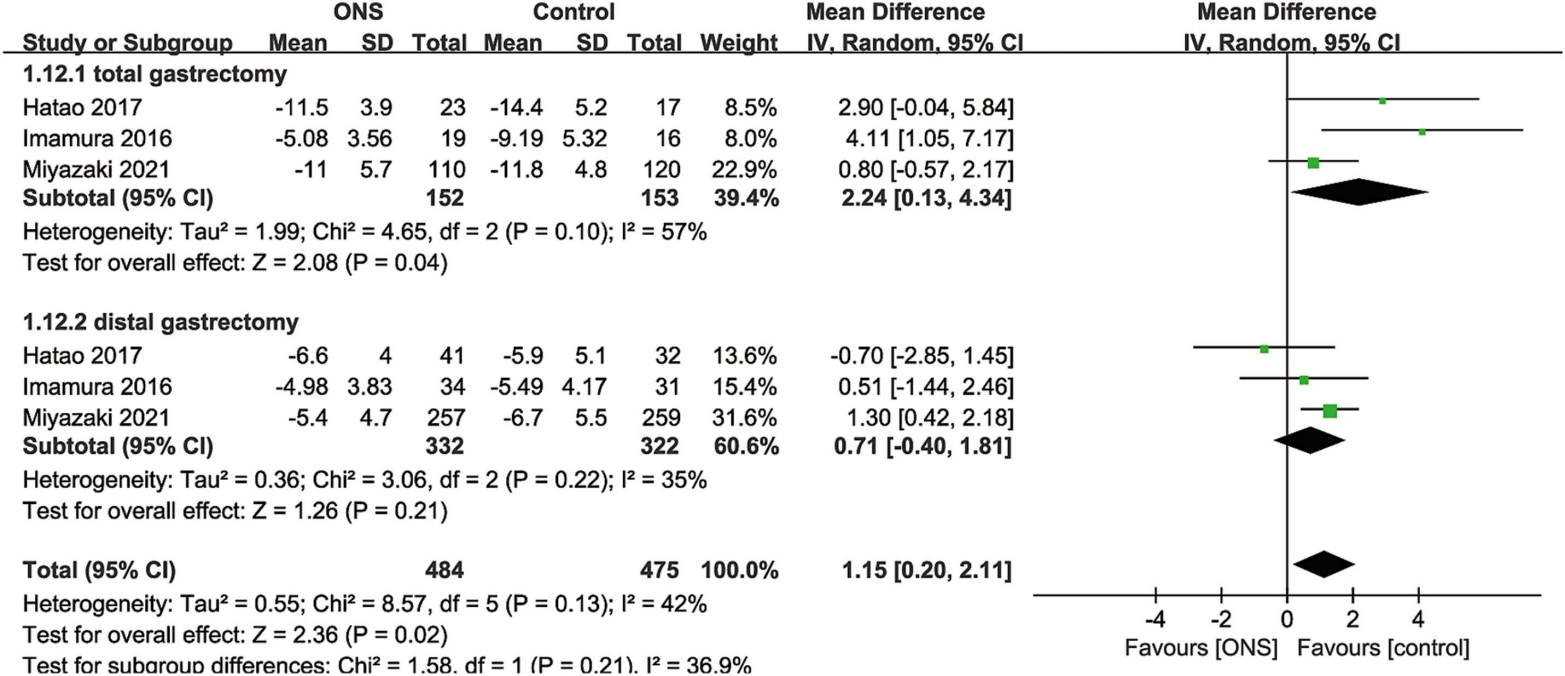
Forest plots of % change of body weight.

Data synthesis of % change of body weight was performed in 3 RCTs including 959 patients (484 ONS versus 475 control) (10, 15, 17). Meta-analysis observed a significant lower % body weight loss in the ONS group (WMD: 1.15; 95% CI: 0.20, 2.11; *p* = 0.02) without significant heterogeneity (*I*^2^ = 42%, *p* = 0.13; Figure 3). Subgroup analysis found that the significance remained in total gastrectomy group (WMD: 2.24; 95% CI: 0.13, 4.34; *p* = 0.04) but disappeared in distal gastrectomy group (WMD: 0.71; 95% CI: −0.40, 1.81; *p* = 0.21; Figure 3).

### Absolute change of body composition

Given the absolute change of body composition between the two groups, including absolute change of skeletal muscle mass and body fat mass, was reported in only one study, we could not perform pooled analysis. Based on the findings of Hatao et al. (15), no significant difference was observed between the ONS and control group for absolute change of skeletal muscle mass and body fat mass both in distal (absolute change of skeletal muscle mass, WMD: −0.20; 95% CI: −0.73, 0.33; *p* = 0.47; absolute change of body fat mass, WMD: 0.10; 95% CI: −1.06, 1.26; *p* = 0.86) and total gastrectomy group (absolute change of skeletal muscle mass, WMD: 0.60; 95% CI: −0.49, 1.69; *p* = 0.29; absolute change of body fat mass, WMD: −0.30; 95% CI: −1.89, 1.29; *p* = 0.72).

### Absolute change of laboratory parameters

In this study, only one RCT (10) reported the absolute change of laboratory parameters between the two groups (mainly including absolute change of albumin, absolute change of total protein, absolute change of total cholesterol, and absolute change of hemoglobin). According to this RCT, no significant difference was observed between the ONS and control group for the any absolute change of laboratory parameters (absolute change of albumin, WMD: −0.03; 95% CI: −0.21, 0.15; *p* = 0.75; absolute change of total protein, WMD: 0.12; 95% CI: −0.18, 0.43; *p* = 0.45; absolute change of total cholesterol, WMD: 5.36; 95% CI: −6.50, 17.22; *p* = 0.37; absolute change of hemoglobin, WMD: 0.69; 95% CI: −0.10, 1.48; *p* = 0.10).

### Adverse events

**Figure 4:**
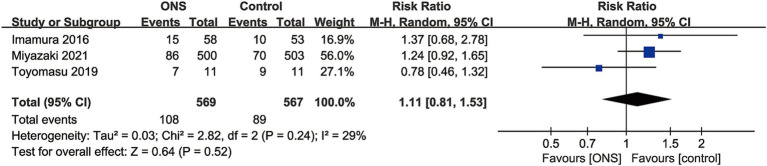
Forest plots of adverse events.

Data of adverse events were available in 3 RCTs with 1,136 patients (569 ONS versus 567 control) (10, 17, 18). No significant difference was found between the ONS and control group for adverse events rate (RR: 1.11; 95% CI: 0.81, 1.53; *p* = 0.52), and no significant heterogeneity (*I*^2^ = 29%, *p* = 0.24) was observed (Figure 4).

### Publication bias

**Figure 5:**
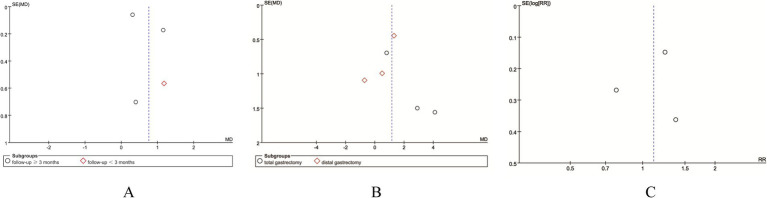
Funnel plots of **(A)** absolute change of body weight, **(B)** % change of body weight, and **(C)** adverse events.

We assessed the potential publication bias through funnel plots and Egger’s regression tests for absolute change of body weight, % change of body weight, and adverse events. No statistical (Egger’s test) or visual (funnel plots) evidence of publication bias was detected for absolute change of body weight (Egger’s test *p* = 0.397; Figure 5A), % change of body weight (Egger’s test *p* = 0.874; Figure 5B), and adverse events (Egger’s test *p* = 0.787; Figure 5C).

### Sensitivity analysis

**Figure 6:**
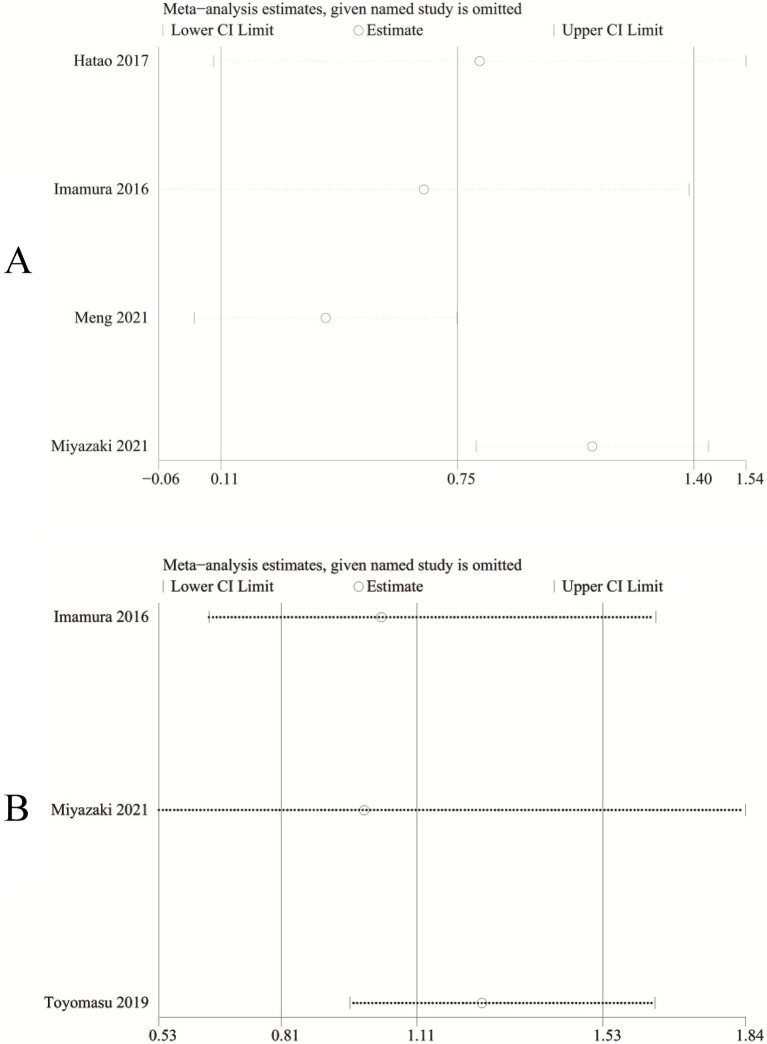
Sensitivity analysis of **(A)** absolute change of body weight and **(B)** adverse events.

We performed sensitivity analysis for the results of absolute change of body weight (Figure 6A) and adverse events (Figure 6B) to assess the effect of each RCT on the total WMD or RR via excluding eligible RCTs one by one. Sensitivity analysis found that the new total RR kept stable after removing of each RCT for adverse events (Figure 6B). However, when we removed the study reported by Imamura et al. in 2016 (10), the pooled analysis of absolute change of body weight changed from significant to nonsignificant (WMD: 0.66; 95% CI: −0.06, 1.38; *p* = 0.07; Figure 6A). In addition, after excluding the data reported by Miyazaki et al. (17), the heterogeneity of absolute change of body weight reduced from 87 to 0%, suggesting that this paper may be the main cause of the significant heterogeneity in the absolute change of body weight.

### GRADE classification

The effects of ONS on the % change of body weight and adverse events were graded as “moderate” quality evidence due to the serious imprecision. However, the effect of ONS on the absolute change of body weight was graded as “low” quality evidence because of the serious inconsistency and serious imprecision observed in this outcome (Table 3).

**Table 3:** GRADE classification of quality of evidence.

Outcome	Metric	Estimate (95% CI)	*I*^2^, *p* value	*p* value of egger’s test	No. of RCTs	Risk of bias	Inconsistency	Indirectness	Imprecision	Publication bias	Plausible confounding	Magnitude of effect	Dose–response gradient	Quality
Absolute change of body weight	MD	0.75 (0.11–1.40)	87%, <0.001	0.397	4	No serious risk	Serious inconsistency	No serious indirectness	Serious imprecision	Undetected	Would not reduce effect	No	No	Low
% change of body weight	MD	1.13 (0.50–1.76)	42%, 0.13	0.874	3	No serious risk	No serious inconsistency	No serious indirectness	Serious imprecision	Undetected	Would not reduce effect	No	No	Moderate
Adverse events	RR	1.21 (0.94–1.55)	29%, 0.24	0.787	3	No serious risk	No serious inconsistency	No serious indirectness	Serious imprecision	Undetected	Would not reduce effect	No	No	Moderate

## Discussion

The continuous loss of body weight after gastrectomy is a symptom correlated to malnutrition, a non-avoidable and fatal problem which related with a damage in postoperative quality of life, decreased function of immune system, and a poor gastric cancer prognosis (28–31). Although several strategies against harmful impact on weight loss caused by gastrectomy have been proposed, significant weight loss associated with a severe malnutrition is still an unsolved problem, notably for patients with gastric cancer after total gastrectomy (17). In a meta-analysis including 13 articles, ONS was observed to significantly improve nutritional intake and some fields of quality of life in malnourished patients suffer from cancer (32). The efficacy of nutritional support through ONS for gastrointestinal diseases in the early period after surgery has also been observed (33). Depending on the degree of preoperative malnutrition, perioperative and postoperative nutritional support could be provided by the simplest means of ONS (34).

In this meta-analysis, we evaluated the efficacy and safety of post-discharge ONS for patients with gastric cancer undergoing gastrectomy. Our results demonstrated that the patients in ONS group had significantly less absolute and % reduction of body weight than those in control (regular diet/dietary advice) group. In addition, compared with the control (regular diet/dietary advice) group, there was no significant increase in the risk of adverse events in the ONS group, suggesting that long-term regular use of ONS after gastrectomy is effective and safe. Results of this meta-analysis are similar to those of previous studies (10, 15–18), which found that post-discharge ONS is an effective and safe nutritional support therapy strategy to improve nutritional parameters after gastrectomy.

Moreover, our subgroup analysis found that the efficacy of ONS remains significant in study with a follow-up time of less than 3 months, while the significance disappeared in studies with a follow-up time of ≥3 months. The time trend of weight loss after gastrectomy may be one of the main reasons for this outcome. It is reported that weigh loss after gastrectomy is time dependent (17). Weight loss that arose after total gastrectomy has been observed to be 15 ~ 20%, of which more than 80% of weight loss occurred during the first 3 months and the remaining 20% of weight loss developed slowly over time (35). This condition may be attributed to the gradually increment of food intake in the early period after gastrectomy and the stabilization of food intake 6 months after gastrectomy (17). Furthermore, subgroup analysis found that the significant efficacy of ONS on the % change of body weight remained in total gastrectomy group but disappeared in distal gastrectomy group. Interestingly, postoperative weight loss was observed to be more serious in total gastrectomy than in distal gastrectomy patients, suggesting that these patients may benefit more from the nutritional improvements provided by ONS (15, 36). On the other hand, the subgroup analysis of total gastrectomy had a significant heterogeneity, which could affect the reliability of the results. In addition, tumor stage and pathological type may also affect the efficacy of ONS. Although the vast majority of gastric cancer patients who undergo gastrectomy are early- to middle-stage patients (I-III), the stages of patients included in different studies are not completely consistent. At the same time, due to insufficient data, we were unable to perform subgroup analysis by tumor stage and pathological type, and it is not yet certain whether the conclusions of this study will be affected by tumor stage and pathological type. Future studies comparing the differences in the absorption and metabolism of ONS between distal and total gastrectomy patients may explain these findings.

The findings in this paper are consistent with the conclusions of the meta-analysis by Choi et al. (20). However, Choi et al.’s meta-analysis focused on the efficacy of perioperative ONS (including short-term application of ONS before and after surgery), but this paper mainly focused on the efficacy of long-term ONS supplementation after surgery for gastric cancer. In addition, due to differences in inclusion criteria, the RCTs published by Toyomasu et al. (18) were included in this study, making the safety assessment of ONS realized in this paper. Furthermore, Choi et al.’s meta-analysis conducted subgroup analysis on energy intake, while this paper conducted subgroup analysis on primary outcomes through follow-up time (≥3 months and < 3 months) and surgical method (distal gastrectomy and total gastrectomy), revealing the potential impact of follow-up time and surgical method on the efficacy of ONS. It provides theoretical support for exploring the optimal suitable population and applicable conditions of ONS. In addition, this study explored the stability of results and potential sources of heterogeneity through sensitivity analysis, and made evidence recommendations for all outcomes through GRADE rating, which is crucial for guideline recommendation of ONS. Therefore, to sum up, the findings of this study mainly confirmed the efficacy and safety of long-term postoperative use of ONS for patients with gastric cancer, and provided the most comprehensive and latest evidence for clinical application of ONS to improve the long-term postoperative nutritional status of patients with gastric cancer.

As an auxiliary means of nutritional supplementation, ONS is mainly used to supplement the various nutrients needed by the human body (37). Its main ingredients include: protein (including whey protein powder, soy protein, etc.), vitamins and minerals (including vitamin C, vitamin B complex, vitamin D, iron, magnesium, calcium, zinc, etc.) and special adult supplements such as fish oil, flaxseed oil, plant extracts, barley grass, spirulina, etc. (38, 39). ONS increases the stimulation of food on mucosal cells while ensuring nutritional balance, which is beneficial to the proliferation of mucosal cells, repairing the mucosal tissue that has atrophied or damaged during the perioperative period as soon as possible, and maintaining the barrier function of the mucosa (40). During ONS, patients have good self-control over the temperature and intake rate of the nutrient solution, so the incidence of adverse reactions such as abdominal distension and diarrhea is low (37). When gastric cancer patients use chemotherapy drugs, normal cells are also damaged to a certain extent, which stimulates the digestive tract to produce a large amount of serotonin, activates the vagus nerve, and is prone to adverse reactions such as nausea, vomiting, and anorexia (38). ONS contain effective active ingredients such as gingerol, which can reduce patients’ gastrointestinal reactions.

## Limitations

We must acknowledge several limitations of this meta-analysis. Firstly, only 2 of 5 included RCTs had low risk in the allocation concealment and only 1 RCT had low risk in the blinding of participants, personnel, and outcome assessment. Secondly, the RCTs included in our study had different intervention (different type of ONS), which may be one of the sources of heterogeneity. Thirdly, most of included studies did not report the specific caloric consumption of the regular diet, and the quantity of ONS consumption was self-recorded by the patients using a custom dietary notebook. In this case, the effects of ONS consumption on the patient’s daily diet and the discrepancy in total dietary energy intake between the two groups remain unclear, which could lead to bias to some extent. Fourthly, all of the included studies were from Asian countries (including Japan and China) and the data of other populations were still deficient. Despite several limitations of this meta-analysis, we conducted the first and largest meta-analysis of RCTs to evaluate the efficacy and safety of post-discharge ONS for patients with gastric cancer undergoing gastrectomy. Results of this meta-analysis validated the superiority of the ONS for the nutritional support of postoperative gastric cancer reported by previous studies. More large-scale, multi-center RCTs are needed to further confirm our findings.

## Conclusion

Pooled analyses revealed that ONS was significantly effective and safe in improving postoperative weight loss for patients with gastric cancer undergoing gastrectomy. Subgroup analysis found that the efficacy of ONS remained significant in total gastrectomy group and study with a follow-up time of less than 3 months, while it seemed to be noneffective in distal gastrectomy group and studies with a follow-up time of ≥3 months. More large-scale, multi-center RCTs are needed to further evaluated the efficacy and safety of post-discharge ONS for patients with gastric cancer undergoing gastrectomy.

## Data Availability

The original contributions presented in the study are included in the article/Supplementary material, further inquiries can be directed to the corresponding author.

## Supplementary material

SUPPLEMENTARY FIGURE S1 Details of the quality evaluation for included RCTs.
