# Supplementary material for: Efficacy and safety of post-discharge oral nutritional supplements for patients with gastric cancer undergoing gastrectomy: a meta-analysis of randomized controlled trials
Source: Front Nutr. 2025 Jan 14;11:1488054. doi: 10.3389/fnut.2024.1488054 (PMC11772097; doi:10.3389/fnut.2024.1488054)
Supplement: SUPPLEMENTARY FIGURE S1 — Details of the quality evaluation for included RCTs. [file Image_1.pdf]

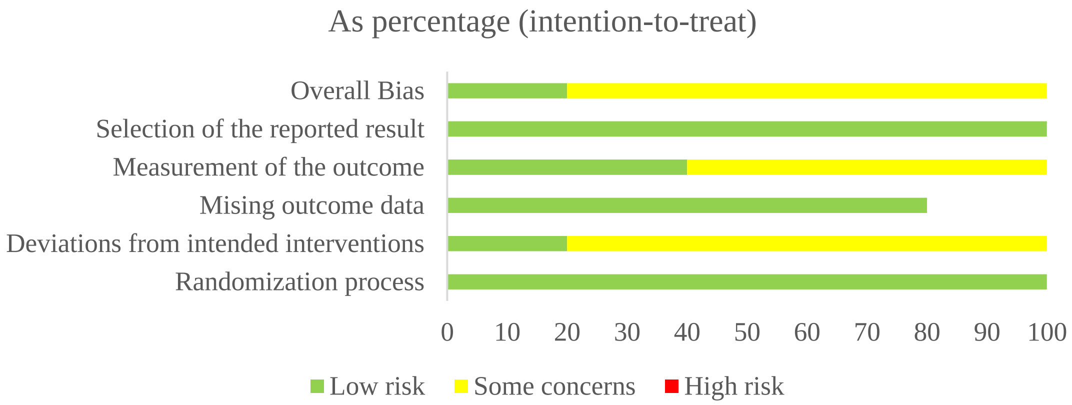
As percentage (intention-to-treat)
Overall Bias
Selection of the reported result
Measurement of the outcome
Mising outcome data
Deviations from intended interventions
Randomization process
0
10
20
30
40
50
60
70
80
90
100
Low risk
Some concerns
High risk

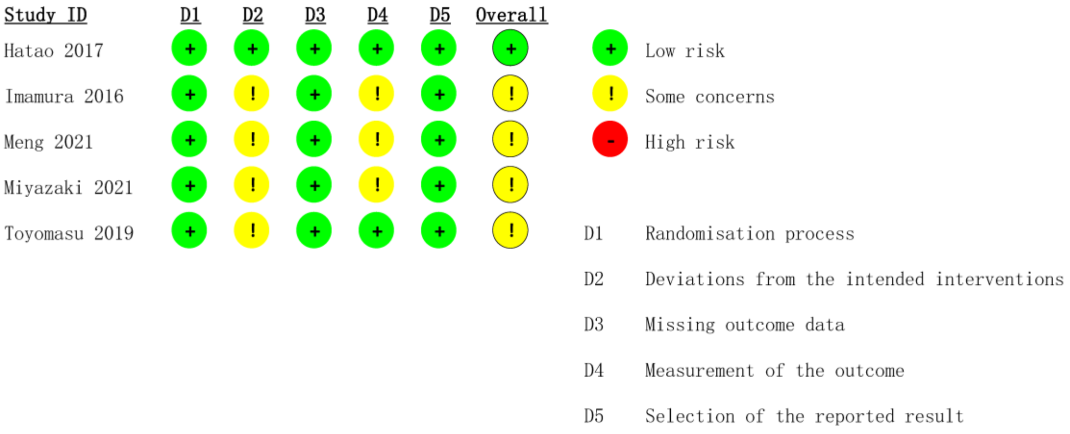
Study ID
D1
D2
D3
D4
D5
Overall
Hatao 2017
Imamura 2016
Meng 2021
Miyazaki 2021
Toyomasu 2019
Low risk
Some concerns
High risk
D1 Randomisation process
D2 Deviations from the intended interventions
D3 Missing outcome data
D4 Measurement of the outcome
D5 Selection of the reported result
